# Metallocene Voltammetric Reference Related to a Normal Hydrogen Electrode

**DOI:** 10.1021/acs.jpclett.4c03390

**Published:** 2025-02-28

**Authors:** Vlastimil Dorčák, Jan Hrbáč, Jiří Janata, Jan Vacek

**Affiliations:** † Department of Medical Chemistry and Biochemistry, Faculty of Medicine and Dentistry, Palacky University, Hnevotinska 3, 775 15 Olomouc, Czech Republic; ‡ Department of Chemistry, Faculty of Science, Masaryk University, 625 00 Brno, Czech Republic; § School of Chemistry and Biochemistry, Georgia Institute of Technology, Atlanta, Georgia 30332-0400, United States

## Abstract

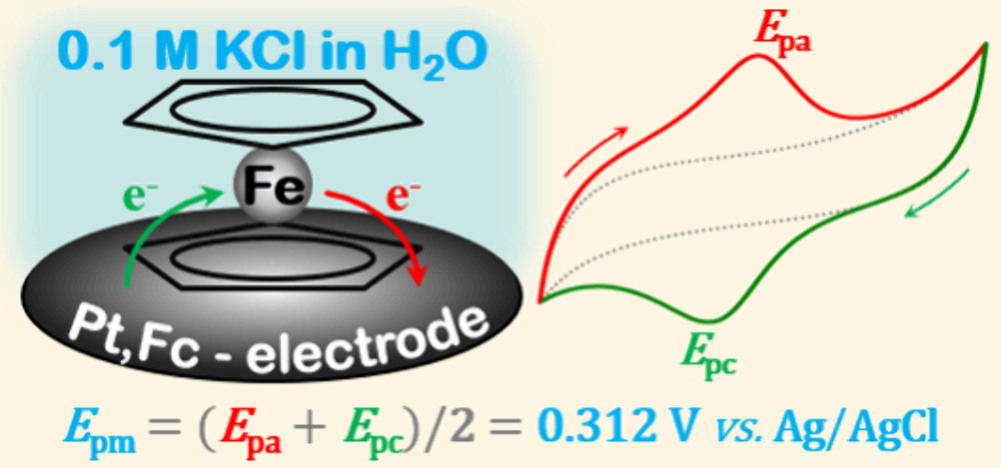

The potentials of electrochemical processes in ideal aqueous media are related to the potential of a normal hydrogen electrode (NHE). However, in non-ideal media, the potentials of a metallocene redox couple are used as a reference. Such measurements with free metallocene in solution are complicated by adsorption and mass transport phenomena and solvation effects. Herein, a platinum electrode with an anchored ferrocene moiety (Pt,Fc) was used for cyclic voltammetric measurements of the potential of ferrocene/ferrocenium (Fc/Fc^+^) redox transformation in not only non-aqueous but, for the first time, aqueous solutions as well. This enabled us to eliminate the aforementioned problems associated with the application of free metallocene molecules in solution and, thus, to relate the midpoint potential (*E*_pm_) of the Fc/Fc^+^ redox couple to a NHE. After elimination of the liquid junction potential in an aqueous 0.1 M KCl solution at 25 °C, the average intraday *E*_pm_ value obtained with freshly prepared Pt,Fc electrodes was found to be 0.312 ± 0.008 V versus the secondary Ag|AgCl electrode. The Pt,Fc electrode can be applied for the standardization of electrochemical measurements and investigation of solvation phenomena at interfaces in non-ideal media.

For many years, ferrocene [C_10_H_10_Fe] has been the subject of research interest in not only organometallic chemistry but also electrochemistry, supramolecular chemistry, materials and analytical chemistry, polymer chemistry, and others.^1^ The unique structural feature of ferrocene was published in 1952.^2^ Immediately afterward, its electrochemical behavior was described using polarography,^3^ followed by a pioneering study with a platinum electrode.^4^ Ferrocene undergoes a single-electron anodic reaction at a potential of around 0.4 V versus a saturated calomel electrode, in which ferrocene (Fc) is converted to a ferrocenium (Fc^+^) moiety. The electrochemical reaction is reversible on common electrode materials, and over the following years, the Fc/Fc^+^ redox couple was gradually established as a redox potential standard (reference) for electrochemical measurements in non-aqueous media,^5^ to “circumvent” the generally known problem with the liquid junction potential of the reference electrodes.^6^

Because the redox potential of Fc changes depending upon the solvent used, new metallocene derivatives have been developed to suppress solvation effects. Thus far, the best known example is decamethyl-Fc but also other permethylated metallocenes, in which the central metal atom is more protected from interactions with the solvent.^1,7^ The problem of solvation increases in electrochemical experiments with mixed solvents and especially in electrolytes containing water. Adsorption processes are also typical for such non-ideal media, which again make it impossible to use the Fc/Fc^+^ redox couple as an internal standard for determining the potential scale. This problem can be overcome by covalently anchoring Fc to the surface of the working electrode by silanization^8^ or using other strategies, such as Schiff base formation, Friedel–Crafts alkylation, or azide–alkyne cycloaddition click reaction.^9^ An original and simple anchoring of vinyl-Fc was recently demonstrated on the surface of a carbon electrode.^10^ However, carbon electrodes suffer from heterogeneity and complex surface chemistry as well as the presence of impurities in the carbon material used for their fabrication^11^ and cannot be used as a universal reference.

Herein, we anchored vinyl-Fc to the surface of a Pt electrode to eliminate or suppress the aforementioned problems associated with the use of free metallocenes in solution. The prepared Pt,Fc electrodes were used for cyclic voltammetric measurements of the Fc/Fc^+^ redox transformation in non-aqueous and also, for the first time, aqueous solutions. The aim of this letter is to introduce a new stable Pt,Fc secondary voltammetric reference electrode applicable for non-ideal media or mixed solvents as an internal redox potential standard. The Pt,Fc electrode enabled us to eliminate the unknown and unpredictable liquid junction potential and, according to a recently reported scheme,^12^ to relate the Fc/Fc^+^ redox couple potential to a normal hydrogen electrode (NHE).

*Pt,Fc Electrode Preparation*. Fc immobilization was performed on a Pt electrode with vinyl-Fc from a 50 mM tetraethylammonium perchlorate/dichloromethane (TEAP/DCM) solution at laboratory temperature. We used a method^10^ originally optimized for a glassy carbon electrode for the immobilization. The cyclic voltammetric anodic electrodeposition pattern for 2 mM vinyl-Fc is shown in Figure 1A. The Pt,Fc electrode prepared in this way was rinsed [acetonitrile (ACN)] and measurement of electron transfer of anchored Fc/Fc^+^ by cyclic voltammetry (CV) was performed in blank 50 mM TEAP/DCM and then also in 50 mM TEAP/H_2_O supporting electrolytes (Figure 2B). For this purpose, we used electrodes that were prepared using vinyl-Fc solutions of different concentrations (0–5 mM); see Figure 1C. The highest Fc/Fc^+^ current responses were obtained for the electrode that was prepared with 2 mM vinyl-Fc (Figure 2D). Under these conditions, the midpoint potential *E*_pm_ = (*E*_pa_ + *E*_pc_)/2 value for the immobilized Fc/Fc^+^ moiety was found to be 0.439 and 0.315 V (versus leak-free Ag|AgCl|3 M KCl) for the organic and aqueous media, respectively (Figure 1C). After the immobilization, Fc/Fc^+^ redox transformations were observed by ∼160 mV less positive potentials compared to free vinyl-Fc; cf. red curve in Figure 1B and corresponding inset. At higher concentrations from 2 to 5 mM vinyl-Fc, the decrease in Fc/Fc^+^ current responses was observed, which is probably due to side reactions, polymerization, and/or electrode passivation events that can suppress the electron transfer (Figure 1D). While no response of Fc/Fc^+^ was indicated at a 10 mM concentration (not shown). According to the obtained Δ*E*_p_ values (*E*_pa_ – *E*_pc_), the reversibility of the immobilized Fc/Fc^+^ redox couple changed with an increasing vinyl-Fc concentration from a quasi-reversible to reversible process in the organic medium, while the opposite was true for the aqueous medium (Figure 1C).

**Figure 1:**
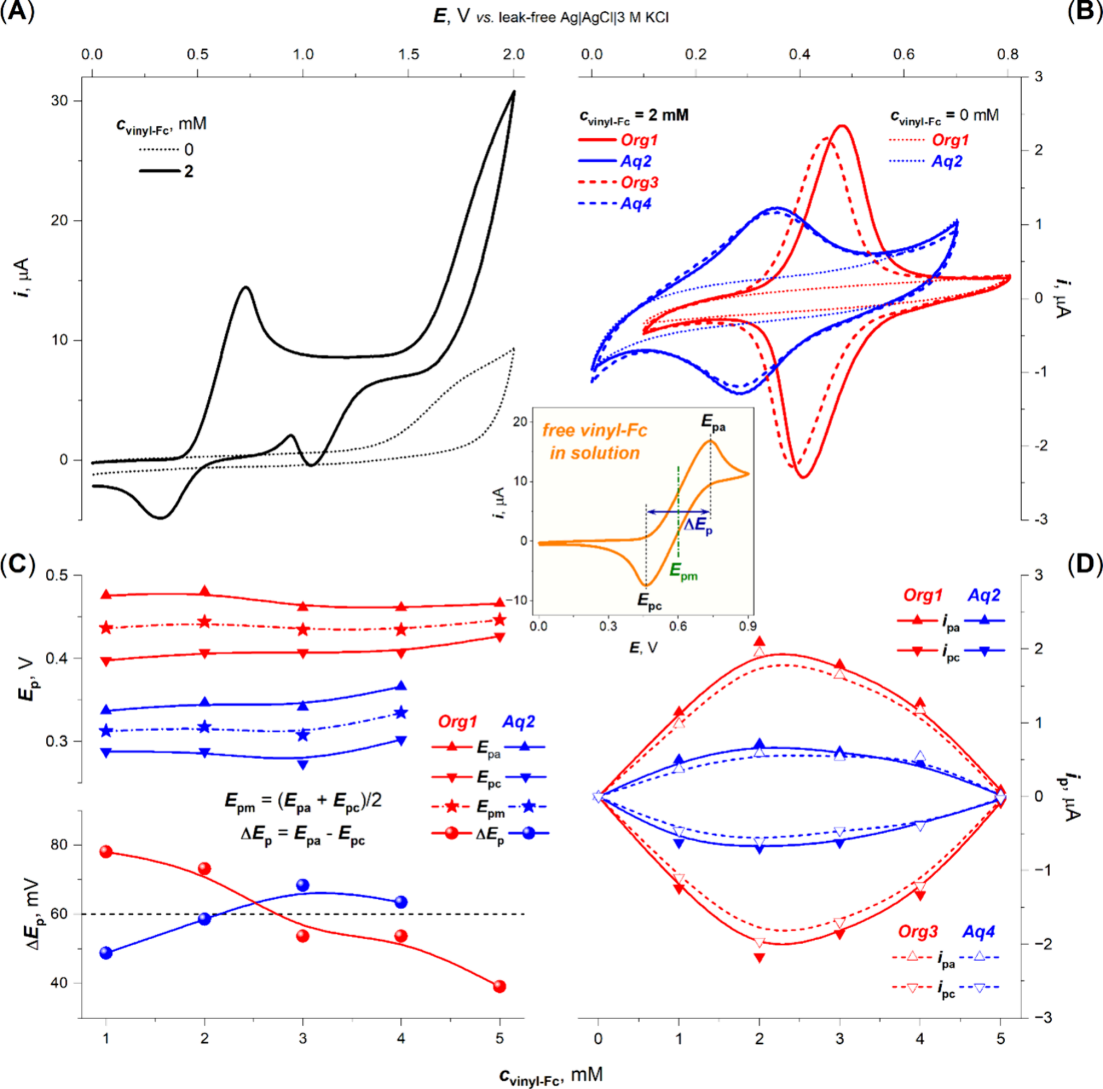
(A) Preparation of the Pt,Fc electrode and (B–D) its cyclic voltammetric characterization in organic and aqueous media. (A) Cyclic voltammograms of vinyl-Fc (at indicated *c*_vinyl-Fc_) in 50 mM TEAP/DCM at a mirror-polished Pt-disk electrode. (B) Steady-state cyclic voltammograms at the Pt,Fc electrode, prepared at indicated *c*_vinyl-Fc_, recorded first in (i) blank 50 mM TEAP/DCM (Org1), then (ii) blank 50 mM TEAP/H_2_O (Aq2) solution, again (iii) blank 50 mM TEAP/DCM (Org3), and again (iv) blank 50 mM TEAP/H_2_O (Aq4) solution. (C) Plotted *c*_vinyl-Fc_ dependences of anodic and cathodic peak potentials (*E*_pa_ and *E*_pa_, respectively), their difference (Δ*E*_p_), and midpoint potential (*E*_pm_) and (D) anodic and cathodic peak currents (*i*_pa_ and *i*_pc_, respectively), evaluated from steady-state cyclic voltammograms. A total of 10 cycles were sufficient to obtain steady-state voltammograms. (Inset) Cyclic voltammogram of free 2 mM vinyl-Fc in 50 mM TEAP/DCM solution at a bare mirror-polished Pt electrode.

**Figure 2:**
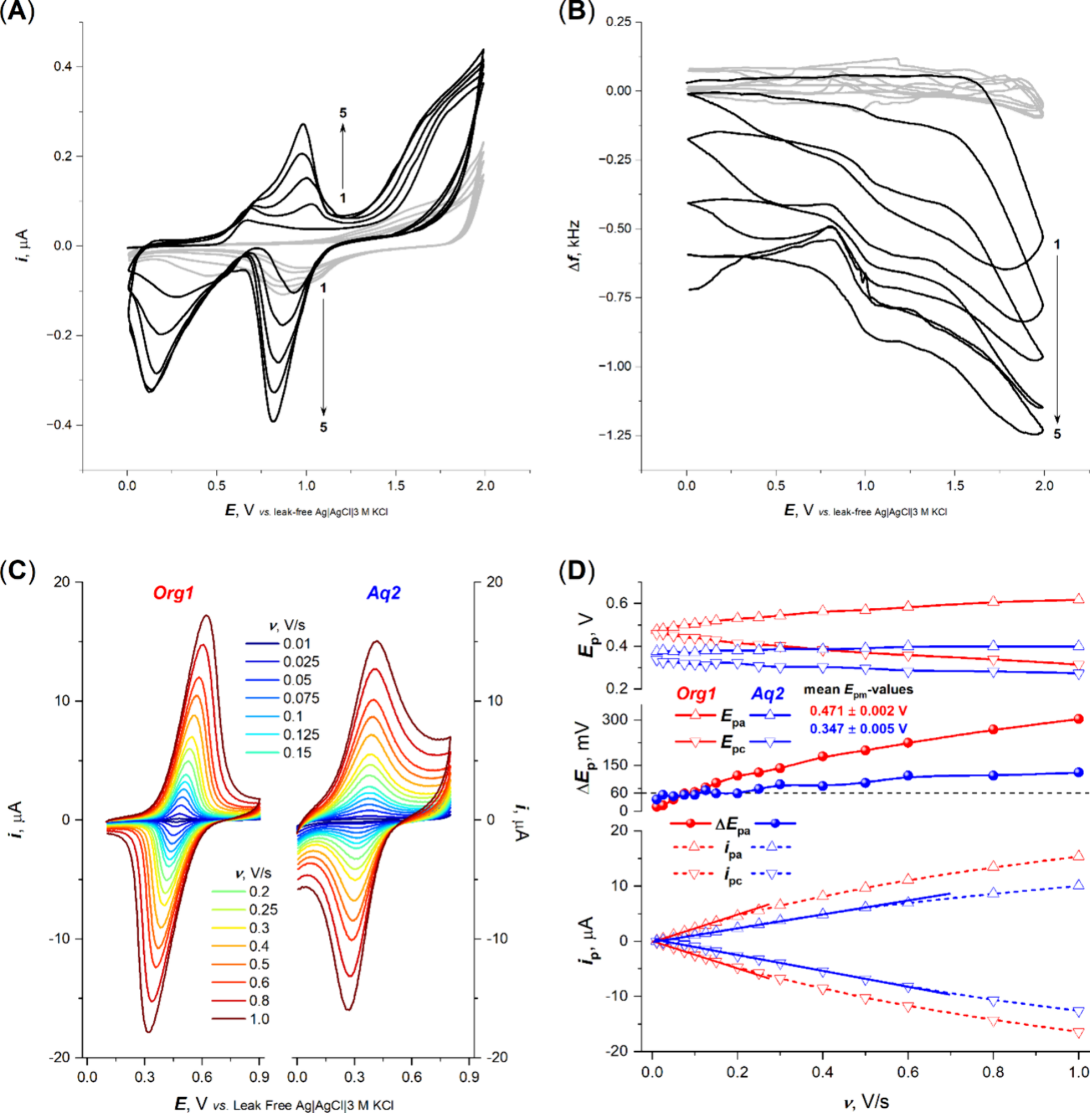
(A and B) EQCM analysis at the Pt,Fc crystal and (C and D) cyclic voltammetric scan rate (ν) dependences at the Pt,Fc electrode. (A) Five consecutive cyclic voltammograms with (B) corresponding EQCM profiles obtained in 50 mM TEAP/DCM with 2 mM vinyl-Fc (black lines); gray curves indicate responses in the blank electrolyte. The arrows indicate the order of the scans. (C) Cyclic voltammograms at the Pt,Fc electrode recorded, at indicated ν values, in first blank 50 mM TEAP/DCM (Org1) and then blank 50 mM TEAP/H_2_O (Aq2) solution. (D) Plotted ν dependences of anodic and cathodic peak potentials (*E*_pa_ and *E*_pc_, respectively), their differences (Δ*E*_p_), and currents (*i*_pa_ and *i*_pc_, respectively) from panel C. All results in panels C and D were gathered with the same Pt,Fc electrode prepared with 2 mM vinyl-Fc.

*Characteristics of the Pt,Fc Electrode*. The electrochemical quartz crystal microbalance (EQCM) confirmed the formation of the vinyl-Fc layer at the Pt electrode (panels A and B of Figure 2). The growth of the layer was observed in not only the first electrodeposition scan but also the consecutive ones. However, the layers prepared using multiple electrodeposition cycles tend toward less reversible voltammetric responses after the transfer into pure supporting electrolytes, indicating the formation of too thick, complex multilayers. Considering the calibration factor of the crystal used (0.105 Hz ng^–1^), the frequency change in the first cycle (620 Hz) corresponds to a mass change of 4.6 μg cm^–2^ or 2.13 × 10^–8^ mol cm^–2^ of vinyl-Fc units. Assuming a vinyl-Fc molecular cross-sectional area of ca. 60 Å^2^ (estimated roughly as a sum of ethylene and cyclopentene cross-sectional areas^13^), the deposition in the first cycle yields ca. 80 monolayers.

The stability of the Fc/Fc^+^ redox couple response was investigated, depending upon the vertex potentials of the electrode polarization. The electrode produces a stable response corresponding to immobilized Fc/Fc^+^ in the range from −0.7 to 1.3 and from −0.5 to 1.15 V (versus leak-free Ag|AgCl|3 M KCl) for DCM and water with 50 mM TEAP media, respectively (not shown). Furthermore, we focused on the effect of the rate of electrode polarization (*v* = 0.01–1 V/s) on the Fc/Fc^+^ electron transfer (Figure 2C). A linear *i*–*v* dependence, for both the anodic and cathodic peaks up to 0.2 and 0.5 V/s (for organic and aqueous media, respectively), indicates that the Fc/Fc^+^ conversion takes place in an adsorbed state. The nonlinear *i*–*v* course at higher *v* values is due to the conversion of deposited Fc/Fc^+^ on the surface of the Pt electrode, not involving further diffusion of the depolarizer from the bulk. The parameter Δ*E*_p_ value indicates the reversibility of the Fc/Fc^+^ conversion up to 0.2 V/s for aqueous media and 0.15 V/s for organic media. At higher values of *v*, the Fc/Fc^+^ process was quasi-reversible, especially for the organic medium (Figure 2D). These observations are in agreement with the previously reported data involving redox processes in thin films.^14^ The average *E*_pm_ value was found to be 0.471 and 0.347 V (versus leak-free Ag|AgCl|3 M KCl) for organic and aqueous media, respectively. Please note that potentials referenced against the leak-free reference electrode do not have any real meaning due to the undefined liquid junction potential [the leak-free Ag|AgCl|3 M KCl reference electrode was used only as a necessity in the preparative experiments and those involving transfer of the Pt,Fc electrode between organic (Org) and aqueous (Aq) media].

*Elimination of the Liquid Junction Potential*. To eliminate a liquid junction, a Ag|AgCl wire was used as the reference for the measurement of Fc/Fc^+^ conversion in 0.1 M KCl (cf. blue and green cyclic voltammograms in Figure 3). This liquid-junction-free experiment would be not feasible without the above-described metallocene electrode with anchored Fc. In this way, we were able to relate obtained potentials to the potential scale of a NHE. The table in Figure 3 shows the intraday averages of *E*_pm_ and *E*_p_ values with standard deviations obtained from five experiments performed with the freshly prepared Pt,Fc electrode at 25 °C in a temperature-controlled cell.

**Figure 3:**
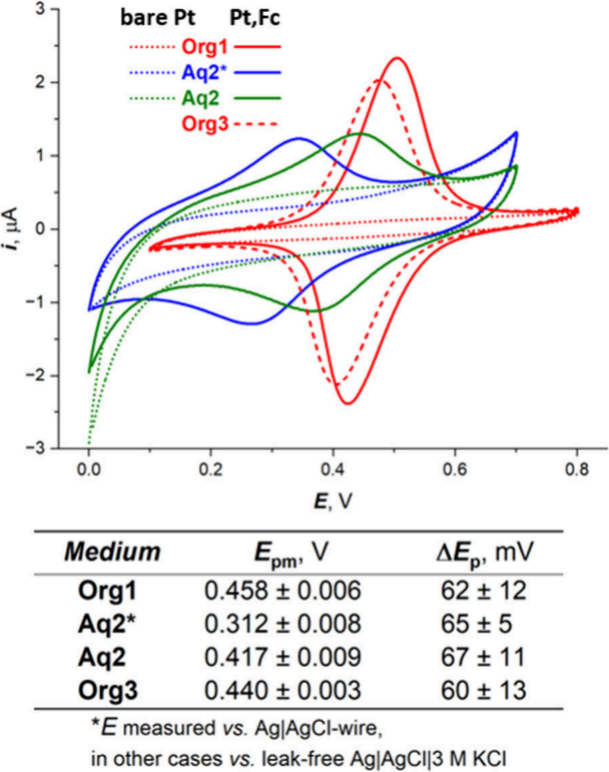
Elimination of the liquid junction potential. Steady-state cyclic voltammograms at the Pt,Fc electrode recorded in first blank solutions of 50 mM TEAP/DCM (Org1), then 100 mM KCl/H_2_O (Aq2), and after that 50 mM TEAP/DCM (Org3). The type of reference electrode used for individual media is specified in the figure legend. The table shows the averages of *E*_pm_ and *E*_p_ values with standard deviations obtained from five experiments performed with the freshly prepared Pt,Fc electrode at 25 °C in a temperature-controlled cell. Other details are the same as those in Figure 1.

The formation of the multilayer deposit of Fc, implemented in this study, does not require any special pretreatments of the Pt electrode. Moreover, Fc anchoring eliminates the adsorption and mass transport phenomena that are typical of experiments with free Fc in solution. The above is also closely related to solvation, which has to be understood before a Pt,Fc electrode can be utilized for measurements in mixed solvents, especially those containing water. We also performed analyses in the presence of free, unanchored 1 mM vinyl-Fc in 50 mM TEAP solution in ACN/water (9:1, v/v), in which we observed a stable Fc/Fc^+^ response over 1 h (not shown). Thus, neither TEAP nor the presence of water interferes with the Fc/Fc^+^ redox couple response (cf. with ref (15), in which Fc moiety degradation was demonstrated in the presence of chloride salts). Using the Pt,Fc electrode, it was also possible to overcome the problems with the accumulation of water, which occurs on the Pt surface during electrochemical measurements.^16^ This effect in our case is shown in Figures 1B and 3, for which the electrode was transferred from water to organic media. This was accompanied by only a small decrease and shift of the Fc/Fc^+^ peak to less positive potentials due to the presence of residual water at the surface of the Pt,Fc electrode. Moreover, we found no formation of precipitates, films, and other undesirable surface reactions that have been previously reported.^17^

In conclusion, the Pt,Fc electrode was used for cyclic voltammetric measurements of the potential of Fc/Fc^+^ redox couple transformation in not only a non-aqueous medium but also aqueous solutions. It enabled us to eliminate the effects of adsorption- and mass-transport-associated phenomena with the application of a metallocene electrode and, thus, relate the *E*_pm_ value of the Fc/Fc^+^ redox couple to a NHE. After elimination of the liquid junction potential in an aqueous 0.1 M KCl solution at 25 °C, the average intraday *E*_pm_ value was found to be 0.312 ± 0.008 V versus the secondary Ag|AgCl electrode. That corresponds to 0.600 ± 0.008 V versus NHE. The Pt,Fc electrode in connection with a “universal reference electrode” can be applied, according to a recently reported scheme,^12^ for the standardization of electrochemical measurements and investigation of solvation phenomena at interfaces in non-ideal media in general.

## Experimental Section

*Reagents*. Dichloromethane (P.A., Lach-Ner), acetonitrile (ACS, VWR Chemicals), tetraethylammonium perchlorate (>99%, Fluka AG), vinyl-Fc (>97%, TCI), KCl (ACS, Sigma), and water (ACS, Thermo Scientific) were used as received.

*Apparatus*. All electrochemical measurements were performed using a μAutolab III analyzer (EcoChemie) connected to a VA-stand 663 (Metrohm) with a three-electrode setup consisting of a Pt-disc working electrode (3.0 mm diameter, 99.95% purity, BASi), a leak-free Ag|AgCl|3 M KCl reference electrode (W3 69-0023, Warner Instruments), and a Pt-wire auxiliary electrode. A Maxtek RQCM system combined with a μAutolab III analyzer was used for electrochemical quartz crystal microbalance measurements.

*Procedures*. A Pt-disc electrode was mirror-polished (with 0.05 μm alumina), washed, sonicated in distilled water, and washed in ACN solution. Finally, vinyl-Fc was directly electrodeposited onto the electrode from a 50 mM TEAP/DCM solution with 1–10 mM vinyl-Fc by one cycle of the potential scanning between 0 and 2 V at a 0.1 V/s scan rate. Cyclic voltammetric measurements at the Pt,Fc electrode were performed in aqueous and organic media with a 50 mM TEAP electrolyte or in a 0.1 M aqueous KCl solution at a 0.1 V/s scan rate (unless stated otherwise). ACN was used to wash the electrodes between transfer from aqueous to organic media and vice versa. All experiments were open to air and performed at laboratory temperature, unless stated otherwise.
